# Is hysterectomy beneficial in radical cystectomy for female patient with urothelial carcinoma of bladder? A retrospective analysis of consecutive 112 cases from a single institution

**DOI:** 10.1186/s12894-019-0461-9

**Published:** 2019-04-29

**Authors:** Haiwen Huang, Bing Yan, Meixia Shang, Libo Liu, Han Hao, Zhijun Xi

**Affiliations:** 1 Department of Urology, Peking University First Hospital, 8 Xishiku Street, Xicheng District, Beijing, 100034 China; 2 Institute of Urology, Peking University, National Urological Cancer Center, 8 Xishiku Street, Xicheng District, Beijing, 100034 China; 3 Department of Urology, Xingtai People’s Hospital, 16 Hongxing Street, Qiaodong District, Xingtai, 054001 China; 4 Department of Medical Statistics, Peking University First Hospital, 8 Xishiku Street, Xicheng District, Beijing, 100034 China

**Keywords:** Radical cystectomy, Urinary bladder neoplasms, Hysterectomy, Female

## Abstract

**Background:**

There is no criterion for determining whether female patients operated with cystectomy would benefit from hysterectomy. This study compares the oncological outcomes between female patients receiving uterus preserving cystectomy (UPC) and uterus excision cystectomy (UEC).

**Methods:**

Retrospective review of 121 female patients with urothelial carcinoma of bladder undergoing UPC (*n* = 63) or UEC (*n* = 49) at a single institute between January 2006 and April 2017. Individual postoperative follow-up plans were performed for patients through outpatient visits. Overall survival (OS) and progression-free survival (PFS) estimates were analyzed using Kaplan-Meier method and multivariable Cox regression.

**Results:**

The median follow-up time was 36 months (interquartile range 16–69). Among patients, 5 (4.1%) had uterus invasion. OS probability (*p* = 0.939) and PFS probability (*p* = 0.565) were similar in two groups. In multivariable Cox regression analysis, hysterectomy was not found to be a predictor of OS (hazard ratio 0.908, 95%CI 0.428–1.924, *p* = 0.801) and PFS (hazard ratio 1.109, 95%CI 0.439–2.805, *p* = 0.826) after adjusting for age, preoperative clinical stage, pathological stage, pathological nodal stage, neoadjuvant/adjuvant chemotherapy, location of the tumor, and surgical margin. No significant difference of overall survival probability was observed in the patients with organ-confined bladder cancer (*p* = 0.675) and in patients with no organ-confined bladder cancer (*p* = 0.695).

**Conclusions:**

The results showed that the rate of uterus invasion was low in patients analyzed in this cohort. It was also found that hysterectomy was not an independent predictor of OS and PFS after radical cystectomy in patients with bladder cancer.

**Electronic supplementary material:**

The online version of this article (10.1186/s12894-019-0461-9) contains supplementary material, which is available to authorized users.

## Introduction

Bladder cancer is the ninth most common cancer worldwide [1]. Despite the 4-fold higher incidence of bladder cancer among males than females, the latter have more advanced tumors at the time of diagnosis [2]. Radical cystectomy is the recommended treatment for recurrent high grade or muscle-invasive bladder cancer. Classical radical cystectomy in women involves en bloc removal of the bladder, entire urethra and adjacent vagina, uterus, distal ureters, and regional lymph nodes [3]. Recently, urological surgeons are considering whether hysterectomy is beneficial for women who undergo radical cystectomy.

The rationales of hysterectomy are the worse prognosis of female patients and in women there is no anatomical barrier in the vesicocervical areas (unlike in men, fascia of Denonvilliers prevent the spread of cancer from bladder and prostate to adjacent rectum) [4]. For patients with advanced urothelial carcinoma of bladder, hysterectomy sufficiently abrogates the tumor. However, previous studies reported that the involvement of the uterus in this condition was only 0.3–12.5% [5–10]. It has also been reported that the most commonly involved gynecologic organ is anterior vagina and not the uterus [5]. Furthermore, preserving the uterus is an important consideration for younger women who desire to retain their fertility, prevent the formation of pelvic prolapse and enterocele [7], as well as prevent the development of postoperative chronic urine retention [11].

Currently, there is no guideline for preserving the uterus during radical cystectomy. Previous studies have explored the risks of uterus involvement in bladder cancer [5, 8, 10]. In the present study, we compared the pathological outcomes and survival rate of women who underwent radical cystectomy with or without hysterectomy to determine whether hysterectomy is beneficial for such patients.

## Patients and methods

### Patient population

**Fig. 1:**
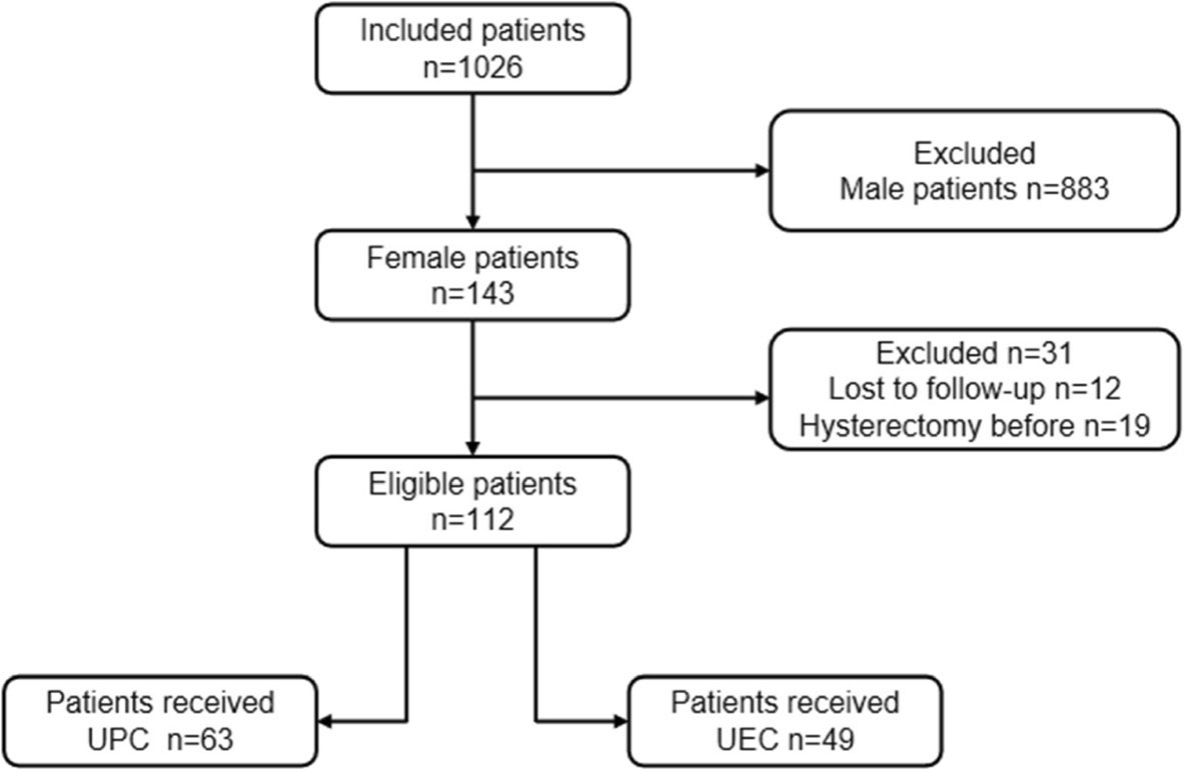
Flowchart of all eligible patients. UPC = uterus preserving cystectomy, UEC = uterus excision cystectomy

This study was approved by the institutional review board of our institute. A total of 1026 patients with urothelial carcinoma of bladder who underwent radical cystectomy (883 males and 143 females) at our institution from January 2006 to April 2017 were analyzed. Among 143 females, 12 patients who underwent hysterectomy before radical cystectomy and 19 patients who were lost to follow-up were excluded (Fig. 1). The indications for radical cystectomy were: T_2-4a_N_0-x_M_0_ tumor, high risk and recurrent non-muscle-invasive tumors and BCG-resistant Tis, as well as extensive papillary disease that cannot be controlled with TUR-Bt and intravesical therapy alone. All patients were diagnosed using computed tomography (CT), magnetic resonance imaging (MRI) or ultrasound (US) and also by pathological examinations including TUR or biopsy sampling. Cisplatin-based combination Neoadjuvant chemotherapy (NAC) was recommended for T_2_-T_4a_N_0_M_0_ bladder cancer patients and adjuvant chemotherapy (AC) was prescribed for patients with pT3/4 and/or pN+ disease if no neoadjuvant chemotherapy had been given, and the patients accepted NAC/AC steadily increased during the study period. The rate of NAC/AC increased from 10.6% in 2006–2011 to 16.9% in 2012–2017. Patients who were suspected with uterine and/or vaginal invasion received cystectomy with hysterectomy. For other patients, the decisions whether hysterectomy was required during radical cystectomy were based on stage of the tumors and patients’ preferences, arrived after adequate discussions between patients and doctors. However, hysterectomy was performed if uterine and/or vaginal invasion was found. In total, 63 patients underwent uterus preserving cystectomy (UPC) whereas 49 patients underwent uterus excision cystectomy (UEC). All surgical complications that occurred within 30 days were classified according to the Clavien-Dindo classification system.

### Outcomes measures

#### Pathological data

Postoperative specimens were analyzed by experienced pathologists of our hospital. Histologic type, tumor grade, tumor and nodal stage, the presence of lymphovascular invasion, perivesical organ and surgical margin status were included in the pathological reports.

#### Oncologic outcomes

Individual postoperative follow-up plans were performed for patients through outpatient visits. During the follow-up period, history, physical examination, laboratory measurements of blood and urine were performed every 3 months in the first year, semi-annually in the second year and annually thereafter. At the same time, a CT scan was performed every 6 months until the third year and annually thereafter. Postoperative disease progression was characterized in terms of local recurrence (takes place in the soft tissues of original surgical site or in LNs), distant recurrence and urothelial recurrences.

### Technique of uterus preserving cystectomy

Uterus preserving cystectomy can be performed though open or laparoscopic approaches. Initially, bilateral pelvic lymphadenectomy was performed. Thereafter, the peritoneum covering uterovesical pouch was incised to expose the posterior wall of bladder to access the trigone. Ligasure was used to transect the bilateral bladder pedicles. The uterine arteries and veins in front of distal ureters were preserved. There were autonomic nerves from the pelvic plexus existing on the posterolateral portion of the rectum and passing caudally on their way to the bladder neck and urethra to run dorsal to the distal ureter [11]. So it was necessary to be careful to dissect along bladder neck and proximal urethral, and avoid using electrocautery as soon as possible at this step. The anterior peritoneum of bladder was incised to access the retropubic space. Suture and ligature the dorsal vein of the clitoris together with the surrounding fascia. All sides of urethra were exposed, ensuring that proximal urethra was removed. This was followed by removal of the specimen and complete urinary diversion.

### Data analysis

The clinicopathologic data of the two groups of patients was categorized based on whether hysterectomy performed. The normality of continuous variables was tested using Kolmogorov-Smirnov test. Independent-Samples t-test was used to compare continuous variables that followed a normal distribution (i.e., the age and time of operation as shown in Table 1). Mann-Whitney U test was used to compare continuous variables that did not follow normal distribution (i.e., BMI, bleeding volume during operation, intraoperative transfusion, postoperative stay and postoperative fasting time as shown in Table 1) between the two groups. For categorical variables, the *x*^2^ test (or Fisher’s exact test) was performed for disordered categorical variables (i.e., ORC/LRC, type of urinary diversion, preoperative clinical stage, pathologic stage, pathologic nodal stage, positive/negative surgery margin, and neoadjuvant/adjuvant chemotherapy [yes/no] as shown in Table 1). Mann-Whitney U test was performed to analyze ordered categorical variables (i.e., ASA score and Clavien-Dindo class as shown in Table 1). Kaplan-Meier curves were used to determine the probability of overall survival and progress free survival among the patients. In addition, to decrease selection bias, the characteristics of survival/dead groups and progression/no-progression groups were compared to eliminate factors may affect the prognosis, after which multivariable Cox regression analysis of all patients was used to test for the effect of hysterectomy on risk of events of death and progress, adjusting for age, preoperative clinical stage (T0/Ta/Tis/T1, T2, T3,T4), pathologic stage (T0/Ta/Tis/T1, T2, T3/T4), pathologic nodal stage (N-, N+), neoadjuvant/adjuvant chemotherapy (yes,no), location of the tumor (posterior wall/trigone, other location) and surgery margin (negative, positive). All analyses were performed using IBM SPSS version23, and all *p* values are two-sided, *p* < 0.05 was considered statistically significant.

**Table 1:** The clinicopathologic characteristics of the patients performed uterus preserving cystectomy and uterus excision cystectomy

	UPC (*n* = 63)	UEC (*n* = 49)	*p*
Age (yr)	67.3 ± 12.2	67.7 ± 9.2	0.855^a^
BMI (kg/m^2^)	22.9 (21.1–26.3)	23.9 (21.7–26.6)	0.205^b^
ASA score			0.961^b^
1	5 (7.9%)	1 (2.0%)	
2	46 (73.0%)	41 (83.7%)	
3	12 (19%)	7 (14.3%)	
ORC	46 (73.0%)	38 (77.6%)	0.582^c^
LRC	17 (27.0%)	11 (22.4%)	
Type of urinary diversion			0.100^c^
Cutaneous ureterostomy	29 (46.0%)	18 (36.7%)	
Ileal conduit	30 (47.6%)	31 (63.3%)	
Orthotopic neobladder	4 (6.3%)	0 (0%)	
Time of operation (mins)	294.8 ± 104.3	296.1 ± 120.0	0.950^a^
Bleeding volume during operation(L)	500 (200–800)	400 (200–650)	0.321^b^
Intraoperative transfusion(L)	200 (0–650)	200 (0–725)	0.762^b^
Clavien-Dindo class			0.061^b^
0	30 (47.6%)	32 (65.3%)	
1	2 (3.2%)	2 (4.1%)	
2	30 (47.6%)	14 (28.6%)	
3	0 (0%)	0 (0%)	
4	1 (1.6%)	0 (0%)	
5	0 (0%)	1 (2%)	
Postoperative stay (days)	11 (8–17)	9 (8–12)	0.076^b^
Postoperative fasting time (days)	5 (3–5)	5 (3–6)	0.537^b^
Location of the tumor			0.399^c^
Posterior wall or trigone	43 (68.3%)	37 (75.5%)	
Other location	20 (31.7%)	12 (24.5%)	
Preoperative clinical stage			0.017^c^
Ta and Tis and T1	18 (28.6%)	12 (24.5%)	
T2	26 (41.3%)	16 (32.7%)	
T3	19 (30.2%)	14 (28.6%)	
T4	0 (0%)	7 (14.3%)	
Pathologic stage			0.613^c^
Ta and Tis and T1	18 (28.6%)	10 (20.4%)	
T2	22 (34.9%)	19 (38.8%)	
T3 and T4	23 (36.5%)	20 (40.8%)	
Pathologic nodal stage			0.002^c^
N0	60 (95.2%)	37 (75.5%)	
N+	3 (4.8%)	12 (24.5%)	
Pathologic grade			1.000^c^
Low grade	7 (11.1%)	5 (10.2%)	
High grade	55 (87.3%)	44 (89.8%)	
Negative margin	60 (95.2%)	47 (95.9%)	1.000^c^
Positive margin	3 (4.8%)	2 (4.1%)	
No neoadjuvant/adjuvant chemotherapy	55 (88.7%)	39 (81.3%)	0.271^c^
Neoadjuvant/adjuvant chemotherapy	7 (11.3%)	9 (18.8%)	

*UPC* uterus preserving cystectomy, *UEC* uterus excision cystectomy, *BMI* Body Mass Index, *ASA* American Society of Anesthesiologists, *ORC* open radical cystectomy, *LRC* laparoscopic radical cystectomy, ^a^Independent-Samples T test, ^b^Mann-Whitney U test, ^c^x^2^ test (or Fisher’s exact test)

## Result

### Clinicopathologic data

This study enrolled 112 patients with urothelial carcinoma of bladder. Among them, 63 underwent uterus preserving cystectomy (UPC) and the rest were treated with uterus excision cystectomy (UEC). The two groups were well-matched with respect to baseline characteristics (Table 1). There was no significant difference between the two patient groups in terms of age, BMI, ASA, method of operation, type of urinary diversion, location of the tumor and neoadjuvant/adjuvant chemotherapy. But patients receiving uterus excision cystectomy had higher preoperative clinical stage (*p* = 0.017). Moreover, there was no difference between two groups in the time of operation, bleeding volume during operation, intraoperative transfusion, postoperative complications, postoperative stay and postoperative fasting time.

Regarding postoperative pathologic characteristics, uterus invasion was found in 5(4.1%) patients and were all from UEC group. There was no significant difference between the two groups with respect to pathologic stage (*p* = 0.613). But higher pathologic nodal stage can be found in UEC group (*p* = 0.002). There was no significant difference in the pathologic grade and surgical margin between the two groups. In the UPC group, two patients had positive margin of urethral, and one patient had positive margin of right ureter. And in the UEC group, one patient had positive margin of vagina, and one patient had positive margin of urethral.

### Oncologic outcomes

**Fig. 2:**
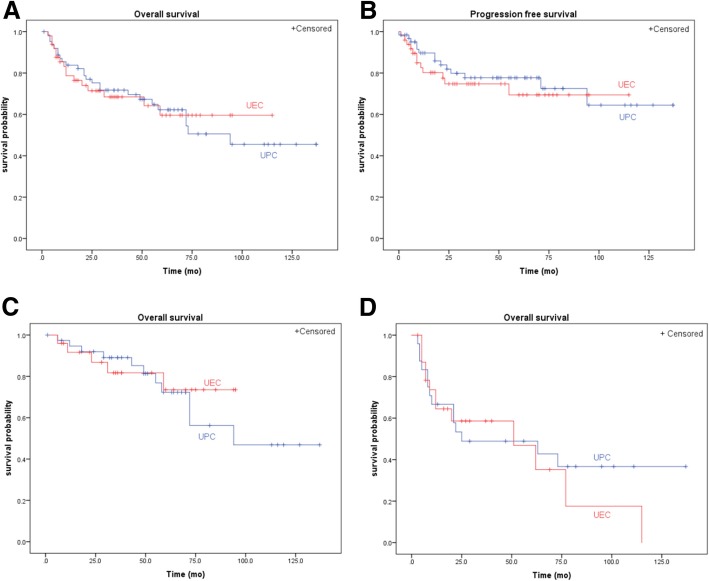
**a** Kaplan-Meier curves of overall survival probability in all patients. The 5-years OS were 0.622 vs 0.596 between two groups, and there were no significant differences in the OS probability (*p* = 0.939). **b** Kaplan-Meier curves of progression-free survival probability in all patients. The 5-years PFS were 0.777 vs 0.694 between two groups, and there were no significant differences in the PFS probability (*p* = 0.565). **c** Kaplan-Meier curves of overall survival probability in organ-confined patients. The 5-years OS were 0.723 vs 0.735 between two groups, and there were no significant differences in the OS probability (*p* = 0.675). **d** Kaplan-Meier curves of overall survival probability in no-organ confined patients. The 5-years OS were 0.489 vs 0.469 between two groups, and there were no significant differences in the OS probability (*p* = 0.695). (UPC = uterus preserved cystectomy, UEC = uterus excision cystectomy)

The median follow-up period for patients was 36 months (interquartile range 16–69). At the last follow-up, the number of deaths in UPC group was 27 (42.9%) and that of UEC group was 20 (40.8%). A total of 15 (23.8%) patients of UPC group and 12 (24.5%) patients of UEC group experienced a progression. Kaplan-Meier curves were used to demonstrate OS probability and PFS probability after UPC and UEC for all patients (shown in Fig. 2a and b). The 5-years OS and PFS were 0.622 and 0.777 in UPC group vs 0.596 and 0.694 in UEC group, respectively. There were no significant differences in the OS probability (*p* = 0.939) and PFS probability (*p* = 0.565) between the two groups.

However, there were more T4 patients and pathological nodal stages in UEC group compared to UPC group. To decrease selection bias, the clinicopathologic characteristics of the survival/dead cases were compared. The analysis revealed that there were significant differences in age (*p* = 0.014), pathologic stage (*p* = 0.014), pathologic nodal stage (*p* = 0.043), and neoadjuvant/adjuvant chemotherapy (*p* = 0.004) between survival and dead cases (Table 2). Similarly, a comparison of the clinicopathologic characteristics of progression and no-progression cases revealed that there were significant differences in pathologic nodal stage (*p* = 0.006), and neoadjuvant/adjuvant chemotherapy (*p* = 0.002) between the two groups (Table 3). Subsequently, multivariable Cox regression analysis was performed to adjust factors that may influence long-term prognosis of patients, and the results are shown in Tables 4 and 5. It was found that hysterectomy was not an independent predictor of overall survival (hazard ratio 0.908, 95%CI 0.428–1.924, *p* = 0.801) and progression-free survival (hazard ratio 1.109, 95%CI 0.439–2.805, *p* = 0.826) after adjusting for age, preoperative clinical stage (T0/Ta/Tis/T1, T2, T3,T4), pathologic stage (T0/Ta/Tis/T1, T2, T3/T4), pathologic nodal stage (N-, N+), neoadjuvant/adjuvant chemotherapy (yes,no), location of tumor (posterior wall/trigone, other location) and surgery margin (negative, positive).

**Table 2:** The clinicopathologic characteristics of the patients of survival group and dead group

	Survival (*n* = 71)	Dead (*n* = 41)	*p*
Age (yr)	65.5 ± 11.5	70.8 ± 9.2	0.014^a^
BMI (kg/m^2^)	23.7 (21.4–26.6)	23.4 (20.8–26.2)	0.570^b^
ORC	50 (70.4%)	34 (82.9%)	0.141^c^
LRC	21 (29.6%)	7 (17.1%)	
Location of the tumor			0.107^c^
Posterior wall or trigone	47 (66.2%)	33 (80.5%)	
Other location	24 (33.8%)	8 (19.5%)	
Pathologic stage			0.014^c^
Ta and Tis and T1	21 (29.6%)	7 (17.1%)	
T2	30 (42.3%)	11 (26.8%)	
T3 and T4	20 (28.2%)	23 (56.1%)	
Pathologic nodal stage			0.043^c^
N0	65 (91.5%)	32 (78.0%)	
N+	6 (8.5%)	9 (22.0%)	
Negative margin	69 (97.2%)	38 (92.7%)	0.159^c^
Positive margin	2 (2.8%)	3 (7.3%)	
No neoadjuvant/adjuvant chemotherapy	66 (93.0%)	29 (72.5%)	0.004^c^
Neoadjuvant/adjuvant chemotherapy	5 (7.0%)	11 (26.8%)	

*BMI* Body Mass Index, *ASA* American Society of Anesthesiologists, *ORC* open radical cystectomy, *LRC* laparoscopic radical cystectomy, ^a^Independent-Samples T test, ^b^Mann-Whitney U test, ^c^χ^2^ test (or Fisher’s exact test)

**Table 3:** The clinicopathologic characteristics of the patients of no progression group and progression group

	No-progression (*n* = 86)	Progression (*n* = 26)	*p*
Age (yr)	69.00 (61.00–76.00)	70.50 (63.75–78.00)	0.370^b^
BMI (kg/m^2^)	23.0 (21.2–26.3)	24.6 (22.3–27.0)	0.100^b^
ORC	65 (75.6%)	19 (73.1%)	0.796^c^
LRC	21 (24.4%)	7 (26.9%)	
Location of the tumor			0.777^c^
Posterior wall or trigone	62 (72.1%)	18 (69.2%)	
Other location	24 (27.9%)	8 (30.8%)	
Pathologic stage			0.159^c^
Ta and Tis and T1	25 (29.1%)	3 (11.5%)	
T2	31 (36.0%)	10 (38.5%)	
T3 and T4	30 (34.9%)	13 (50.0%)	
Pathologic nodal stage			0.006^c^
N0	79 (91.9%)	18 (69.2%)	
N+	7 (8.1%)	8 (30.8%)	
Negative margin	83 (96.5%)	24 (92.3%)	0.329^c^
Positive margin	3 (3.5%)	2 (7.7%)	
No neoadjuvant/adjuvant chemotherapy	79 (91.9%)	17 (65.4%)	0.002^c^
Neoadjuvant/adjuvant chemotherapy	7 (8.1%)	9 (34.6%)	

*BMI* Body Mass Index, *ASA* American Society of Anesthesiologists, *ORC* open radical cystectomy, *LRC* laparoscopic radical cystectomy, ^a^Independent-Samples T test, ^b^Mann-Whitney U test, ^c^χ^2^ test (or Fisher’s exact test)

**Table 4:** Univariable/Multivariable Cox regression analysis of variables associated with overall survival after cystectomy

Variable	univariable cox regression analysis			multivariable cox regression analysis		
	Hazard Ratio	95% CI	*p*	Hazard Ratio	95% CI	*p*
Age (continuous)	1.034	1.003–1.067	0.034	1.027	0.994–1.061	0.108
Preoperative stage			0.113			0.927
Ta and Tis and T1	–	referent	–	–	Referent	–
T2	1.317	0.552–3.141	0.535	0.814	0.281–2.359	0.705
T3	2.496	1.064–5.851	0.035	1.050	0.355–3.100	0.930
T4	2.608	0.683–9.951	0.161	0.862	0.174–4.264	0.856
Pathologic stage			0.005			0.129
Ta and Tis and T1	–	referent	–	–	referent	–
T2	1.375	0.530–3.568	0.513	1.315	0.456–3.792	0.613
T3 and T4	3.341	1.427–7.822	0.005	2.508	0.876–7.174	0.087
Pathologic nodal stage						
N0	–	referent	–	–	Referent	–
N+	2.725	1.285–5.778	0.009	1.896	0.791–4.544	0.152
No chemotherapy	–	referent	–	–	Referent	–
Chemotherapy	2.979	1.486–5.973	0.002	1.860	0.848–4.081	0.121
Location of the tumor						
other location	–	referent	–	–	Referent	–
posterior wall/trigone	2.028	0.934–4.402	0.074	1.902	0.834–4.339	0.126
Negative margin	–	referent	–	–	Referent	–
Positive margin	3.938	1.177–13.172	0.026	1.463	0.381–5.628	0.580
No hysterectomy	–	referent	–	–	Referent	–
Hysterectomy	0.976	0.520–1.832	0.976	0.908	0.428–1.924	0.801

**Table 5:** Univariable/Multivariable Cox regression analysis of variables associated with progression free survival after cystectomy

Variable	univariable cox regression analysis			multivariable cox regression analysis		
	Hazard Ratio	95% CI	*p*	Hazard Ratio	95% CI	*p*
Age (continuous)	1.024	0.986–1.064	0.216	1.013	0.971–1.056	0.556
Preoperative stage			0.168			0.437
Ta and Tis and T1	–	referent	–	–	referent	–
T2	2.011	0.630–6.419	0.238	0.983	0.236–4.102	0.981
T3	3.516	1.114–11.099	0.032	1.529	0.369–6.341	0.558
T4	1.509	0.167–13.609	0.714	0.286	0.025–3.296	0.316
Pathologic stage			0.055			0.541
Ta and Tis and T1	–	referent	–	–	referent	–
T2	2.904	0.792–10.654	0.108	2.003	0.460–8.721	0.355
T3 and T4	4.600	1.300–16.270	0.018	2.419	0.507–11.543	0.268
Pathologic nodal stage						
N0	–	referent	–	–	Referent	–
N+	4.372	1.883–10.150	0.001	4.067	1.424–11.610	0.009
No chemotherapy	–	referent	–	–	Referent	–
Chemotherapy	4.705	2.075–10.670	<0.001	2.950	1.131–7.694	0.027
Location of the tumor						
other location	–	referent	–	–	Referent	–
Posterior wall/trigone	1.004	0.436–2.313	0.992	0.737	0.292–1.859	0.518
Negative margin	–	referent	–	–	Referent	–
Positive margin	3.369	0.778–14.584	0.104	1.500	0.253–8.904	0.656
No hysterectomy	–	referent	–	–	Referent	–
Hysterectomy	1.254	0.578–2.718	0.567	1.109	0.439–2.805	0.826

Furthermore, patients with organ-confined tumors (≤pT2bN0M0) were used in the survival analysis and those with on-organ confined tumors were used for sensitivity analysis to eliminate inclusion bias. and no significant difference of overall survival probability was observed in the patients with organ-confined bladder cancer (*p* = 0.675) and in patients with no organ-confined bladder cancer (*p* = 0.695). (Fig. 2c and d).

## Discussion

Currently, radical cystectomy is the mainstream treatment for recurrent high grade or muscle-invasive bladder cancer. Unlike in male patients, radical cystectomy in female patients includes total anterior pelvic exenteration encompassing the bladder, urethra, anterior vagina and uterus. This type of resection is recommended for patients with vaginal or uterus invasion. However, uterus invasion is rare according to the prevailing literature on postoperative pathological data. Among 160 female patients who underwent radical cystectomy, Gregg et al. found that 20 (12.5%) had uterus invasion [5], which is the highest rate reported so far (0.3–7.5%) [6–10]. In our cohort, among 112 patients who underwent radical cystectomy, only 5 (4.5%) had uterus invasion. According to Kluth, the prognosis of female patients is poorer compared to that of men, further explaining why resection is necessary for female patients. Moreover, it was stated that female gender was an independent risk factor for death from this disease (hazard ratio = 1.17 [range 1.05 to 1.31], *p* = 0.005) [12]. From the anatomical perspective, in women, there is no natural anatomical barrier between the bladder and uterus to prevent tumor invasion [4]. Therefore, majority of urological surgeons hold the view that hysterectomy seems to be a reasonable part of cystectomy, especially for postmenopausal women and those who no longer have the desire to preserve their fertility.

Due to technological advancement of endoscopy technology and the duration of application, many urological surgeons prefer minimally invasive surgery in the management of bladder cancer [13]. In present study, 28 (25%) patients received laparoscopic radical cystectomy (LRC), and during the study period, more patients chose minimally invasive surgery technique. The number of patients who received LRC increased from 14.9% in 2006–2011 to 32.3% in 2012–2017. This is because urological surgeons prefer surgical approaches that improve patients’ postoperative quality of life. For this reason, the use of pelvic organ-preserving techniques has increased tremendously. The need to understand the pelvic structure and improve surgical techniques has driven the emergence of new techniques, such as those aimed at preserving neurovascular bundle, vagina, uterus or variations of any the stated techniques [14]. In present study, 63(56.3%) patients received uterus preserved cystectomy.

Uterus sparing radical cystectomy decreases the rate of sexual dysfunction, preserves fertility and improves the voiding function of orthotopic neobladder [15]. However, in this study, 9 patients were below 50 years old, but we did not ascertain the number of those who wanted to retain their fertility. Nevertheless, not many patients wanted to retain their fertility, in fact many patients chose uterus preserving surgery to avoid losing another organ. Classic radical cystectomy may damage neurovascular bundles along the lateral vaginal wall, which may compromise sexual arousal and orgasm. Female Sexual Function Index can be preserved if autonomic nerves are left intact, but it can be severely diminished when such nerves are removed [16]. For patients receiving orthotopic neobladder, leaving the uterus and vaginal intact will provide proper support to the neobladder and prevent herniation of posterior wall of the pouch through the vaginal. This decreases the rate of postoperative chronic urinary retention and hence improve the voiding function [17, 18]. In this study, a high percent of patients received cutaneous ureterostomy diversion and only 4 patients chose orthotopic neobladder for urinary diversion. Yet, we were alert to the fact that ileal conduits and neobladder reconstruction are better options, especially for patients with a lower stage of disease. As more laparoscopic operations were performed in our institution, more orthotopic neobladder were applied.

Some previous study investigated the risks of uterus invasion to determine which group of patients is likely to benefit from uterus preserving cystectomy. A study by Varkarakis performed in Austria, retrospectively reviewed 54 women who had clinical organ confined transitional cell bladder cancer. In their study, preoperative risk factors could not be identified, although 3 cases of tumor invasion to internal genitalia (1 uterus and 2 vaginal) were observed in the dome and base of bladder [7]. Choi et al. reported that tumor location of the trigone or bladder neck at TUR-Bt, maximum tumor size ≥4.8 cm at CT, and hydronephrosis at CT were independent predictors of female organ involvement [8]. A study by Ali-El-Dein indicated that high grade of the bladder tumor and positive lymph node status were positive predictors of secondary gynecologic organ involvement [10]. Gregg et al. stated that lack of trigonal or bladder floor tumor, intraoperative palpable posterior mass, and clinical lymphadenopathy were associated with absence of female pelvic organ involvement. Hence, unifocal, organ-confined tumors (≤cT2b) away from the bladder neck, trigone, and bladder base seem to be suited for reproductive organ-sparing radical cystectomy [15]. In this study, there was no definite indication for uterus-sparing radical cystectomy. Hysterectomy was performed in radical cystectomy based on the consensus between doctors and patients. Hysterectomy was also performed in cases where preoperative examinations revealed a suspected uterine invasion or when uterine invasion was found during intraoperation. In present study, one patient, who had no uterine invasion from preoperative examination, was changed to uterus excision cystectomy because uterine invasion was found during operation. It should be noted that patients in the two groups were matched in terms of age, BMI and ASA, and the decisions had no relationship with the surgeons’ preference (data not show).

As a treatment for malignant tumors, the main goal of radical cystectomy is to achieve oncological control. This study involved a long-term follow-up duration to assess the oncological outcomes after UPC. Among 112 female patients with bladder cancer, 63 (56.3%) were operated with UPC and 49 (43.7%) received UEC. No significant differences were observed between two groups in overall survival and progression-free survival rates. However, the group of UEC, comprising more cases of T4 and more cases with higher pathological nodal stage, whose prognosis was supposed to be worse than that of the group of UPC. Therefore, to reduce selection bias, we used multivariable Cox regression analysis to adjust for the factors that may affect the patients’ prognosis— age, preoperative clinical stage (T0/Ta/Tis/T1, T2, T3,T4), pathologic stage (T0/Ta/Tis/T1, T2, T3/T4), pathologic nodal stage (N-, N+), neoadjuvant/adjuvant chemotherapy (yes,no), location of the tumor (posterior wall/trigone, other location), and surgery margin (negative, positive). The results showed that hysterectomy was not an independent predictor of overall survival (hazard ratio 0.908, 95%CI 0.428–1.924, *p* = 0.801) and progression-free survival (hazard ratio 1.109, 95%CI 0.439–2.805, *p* = 0.826). Further analysis revealed that, in patients with organ-confined tumors (≤cT2bN0M0), there were no significant differences in overall survival (*p* = 0.675) and progression-free survival (*p* = 0.985) between UPC and UEC groups. Hence, we infer that hysterectomy is not suitable for female patients with organ-confined bladder cancer. Similarly, for patients with advanced tumor, there were no significant differences in the overall survival and progression-free survival between two groups. We postulate that hysterectomy may not be effective in patients with advanced tumor because of poor prognosis. However, this concept need to be clarified in further studies. In this study, the 5-year OS was 0.622 and 0.596 in UPC and UEC groups, respectively, and was slightly lower compared to 65–83% reported in previous studies [14]. This was attributed to the lower percent of patients receiving neoadjuvant/adjuvant chemotherapy in this study. In our case, many patients refused chemotherapy because of its adverse events.

There are some limitations to consider in this study. A key limitation is that this is a retrospective design with some inherent selection bias. In addition, the decision whether to perform hysterectomy in radical cystectomy was based on discussions between doctors and patients. Application of uterine preserving surgery was not based on clear guidelines. Patients with advanced tumors were more likely to choose hysterectomy. Therefore, this study may have some selection biases and confounding biases. We adopted a multivariable Cox regression analysis to adjust for factors that may affect patients’ prognosis instead of a Kaplan-Meier analysis with matched groups because the lower prevalence of the bladder cancer in women contributed to the relatively small sample size. Moreover, due to patients’ privacy and the retrospective nature of this study, we did not include other outcomes such as changes in sexual and urinary function, rate of prolapse, and postoperative fertility.

## Conclusion

The results showed that the rate of uterus invasion was low in patients analyzed in this cohort. It was also found that hysterectomy was not an independent predictor of OS and PFS after radical cystectomy in patients with bladder cancer.

## Additional file

Additional file 1: Information of the patients. This data includes relevant information of all patients enrolled in this study, including Clinicopathologic data and oncologic outcomes. (XLSX 24 kb)

## Abbreviations

AC: Adjuvant chemotherapy
ASA: American Society of Anesthesiologists
BMI: Body Mass Index
CT: Computed tomography
LRC: Laparoscopic radical cystectomy
MRI: Magnetic resonance imaging
NAC: Neoadjuvant chemotherapy
ORC: Open radical cystectomy
OS: Overall survival
PFS: Progression-free survival
UEC: Uterus excision cystectomy
UPC: Uterus preserving cystectomy
US: Ultrasound
